# Unraveling the role of chemokines in cutaneous T-cell lymphoma: expression levels at different stages

**DOI:** 10.3389/fimmu.2025.1646669

**Published:** 2025-08-08

**Authors:** Alba Guglielmo, Corrado Zengarini, Federica Tugnoli, Pier Luigi Zinzani, Alessandro Borghi, Monica Corazza, Michelangelo La Placa, Bianca Maria Piraccini, Alessandro Pileri

**Affiliations:** ^1^ Section of Dermatology and Infectious Diseases, Department of Medical Sciences, University of Ferrara, Ferrara, Italy; ^2^ Department of Medical and Surgical Sciences, University of Bologna, Bologna, Italy; ^3^ Dermatology Unit, IRCCS Azienda Ospedaliero-Universitaria di Bologna, Bologna, Italy; ^4^ Istituto di Ematologia “Seràgnoli”, IRCCS Azienda Ospedaliero-Universitaria di Bologna, Bologna, Italy

**Keywords:** chemokines, receptors, mycosis fungoides, Sézary syndrome, cutaneous lymphoma, receptor, ligand, lymphokine

## Abstract

Mycosis fungoides (MF) and Sezary syndrome (SS) are the most prevalent cutaneous T-cell lymphomas, classified separately in the 2022 WHO Classification due to their distinct features. Despite advances, the mechanisms underlying disease progression—from early patch and plaque lesions to advanced tumor stages—remain incompletely understood. Chemokines and their receptors play crucial roles in the migration and survival of malignant T cells, influencing tissue invasion, immune evasion, and dissemination. This review highlights the altered expression of chemokine receptors like CCR4, CCR7, CCR8, CCR10, CXCR3, and CXCR4 in MF/SS and their contribution to disease evolution. It also explores the transition from a Th1 to a Th2 immune profile, linked to tumor progression. The dual role of chemokines in physiology and pathology is examined, with emphasis on their therapeutic potential in CTCL.

## 1 Introduction

Mycosis fungoides (MF) and Sezary syndrome (SS) are the two most common cutaneous T-cell lymphomas (CTCLs). In the 2022 WHO Classification of Hematopoietic Tumors (1), these entities have been assigned to separate chapters, reflecting growing recognition of their distinct biological and clinical characteristics. However, our knowledge is far from a thorough comprehension of the pathobiology.

The progression of early-stage MF—initially characterized by patch and plaque lesions—to more advanced stages featuring tumor lesions is accompanied by marked changes in clinical presentation, histology, and immunophenotype. The mechanisms driving this progression are currently the subject of investigation (2). Recent studies have highlighted the pivotal role of chemokines and their receptors in regulating homing and chemotactic processes, which are essential for tissue invasion and metastasis. Of particular interest is the loss of epidermotropism—characteristic of early-stage MF—which gives way to deeper dermal invasion in tumor-stage lesions, often followed by lymph node involvement and hematogenous dissemination (3).

Chemokines are small signaling proteins that can be categorized into four major subfamilies—CXC, CC, C, and CX3C—based on the arrangement of conserved cysteine residues near their amino terminus (4).

CC chemokines (e.g., CCL17, CCL22, CCL27) predominantly attract monocytes, T cells, and eosinophils via receptors such as CCR4 and CCR10. CXC chemokines (e.g., CXCL9, CXCL10, CXCL12) mediate neutrophil and lymphocyte recruitment through CXCR3 and CXCR4. CX3CL1, the only known CX3C chemokine, binds to CX3CR1 and plays a unique role in leukocyte adhesion and migration. C-type chemokines (e.g., XCL1) act through XCR1 and have more restricted functions. This structural classification underscores specific receptor-ligand interactions, though the chemokine system is known for its functional redundancy and cross-reactivity (5). Indeed, chemokine receptors are promiscuous: a single chemokine can interact with multiple receptors, leading to different immune outcomes, such as the recruitment of T cells versus eosinophils (6, 7). Chemokines not only mediate chemotaxis but also participate in the activation of both innate and adaptive immune responses. Several chemokines and their receptors exhibit tissue-specific expression. In the skin, molecules such as CXCL8, CXCL9, CXCL10, CCL11, CCL17, CCL26, and CCL27 are expressed and contribute to leukocyte recruitment via the lymphatic and vascular systems. Additional chemokines, including CCL21, CXCL12, and CX3CL1, also play key roles in this process. Disruption of the local chemokine environment has been implicated in the pathogenesis of various skin disorders. In the context of CTCL, chemokine receptors not only facilitate the skin-homing of malignant T cells but also support their survival and resistance to apoptosis by activating prosurvival signaling pathways. Aberrant expression of receptors such as CCR4, CCR7, CCR10, CXCR3, and CXCR4 has been observed in MF and SS and is thought to contribute to disease progression, lymph node dissemination, and immune evasion (2). Changes in cytokine and chemokine profiles in MF/SS may influence the transition from early to advanced disease stages. This shift is often characterized by a switch from an anti-tumor, Th1-dominated immune response to a Th2-driven, immunosuppressive phenotype (8–12). This narrative review explores the involvement of chemokines in MF and SS, emphasizing the role of their receptors and evaluating their potential as therapeutic targets. To aid comprehension, we distinguish between their physiological and pathological roles.

## 2 The expression and functional role of chemokine receptors in mycosis fungoides and Sézary syndrome

### 2.1 CC chemokine receptor 4

#### 2.1.1 Physiological role of CCR4

CC chemokine receptor 4 (CCR4), which binds the ligands CCL22 and CCL17, is expressed on effector regulatory T cells (Tregs), which exert potent immunosuppressive functions and play a key role in dampening antitumor immune responses. CCL17, also known as thymus and activation-regulated chemokine (TARC), is a ligand for both CCR4 and CCR8, and functions primarily to recruit immune cells expressing these receptors. It is secreted by dendritic cells, fibroblasts, plasma cells, and endothelial cells. The expression of CCL17 is upregulated by TGF-β, TNF-α, IL-4, and IL-13, and is enhanced in the context of low CD40 ligand expression. Therefore, CCL17 is classified as a T-helper 2 (Th2)-type chemokine. Conversely, IFN-γ and IL-10 negatively regulate its production (13, 14). Serum CCL17 levels have been shown to correlate with disease activity in various inflammatory and neoplastic skin diseases, including atopic dermatitis, bullous pemphigoid, and CTCL. Notably, CCR4—the receptor for CCL17—is also expressed on neoplastic T cells in CTCL, particularly in MF and SS (6, 15). CCL22 contributes to the regulation of T cell immunity by recruiting Tregs into the tumor microenvironment and facilitating dendritic cell–Treg (DC–Treg) interactions within lymph nodes (16). Under physiological conditions or in inflammatory disorders, CCR4 is expressed by approximately 20–30% of CD4^+^ T cells and 5–8% of CD8^+^ T cells in peripheral blood. In benign inflammatory dermatoses such as atopic dermatitis and psoriasis, skin-infiltrating lymphocytes frequently express CCR4 (3, 17–19). Shono et al. reported that CCR4 expression was more frequent in atopic dermatitis than in CTCL; however, the intensity of CCR4 expression was lower in all CCR4-positive atopic dermatitis cases compared to those with CTCL (20).

#### 2.1.2 CCR4 in mycosis fungoides and Sézary syndrome

In MF/SS, CCR4 binds CCL17 produced by dendritic and endothelial cells in lesional skin. This interaction contributes to the recruitment of neoplastic CD4^+^ T cells, their interaction with dendritic cells, and their subsequent trafficking into the circulation (20–22). Elevated serum levels of CCL17 have been associated with increased disease activity in MF and SS (15, 23). It has been hypothesized that CCL17 expression by Langerhans cells, endothelial cells, and fibroblasts facilitates the recruitment of malignant T cells and Th2-polarized cells to the skin, contributing to the Th2-skewed tumor microenvironment typical of advanced CTCL (9, 10, 12, 21, 24). Multiple studies have reported that serum CCL17 levels are significantly higher in patients with tumor-stage MF compared to those with patch or plaque lesions. Additionally, high CCL17 levels have been positively correlated with elevated serum lactate dehydrogenase (LDH)—a marker of disease burden—further supporting its role as a biomarker of disease severity and progression (15, 25). CCR4 is also widely expressed on tumor cells in adult T-cell leukemia/lymphoma and other peripheral T-cell lymphomas. In the study by Kallinich et al., chemokine and chemokine receptor expression was analyzed in both malignant T cells and cells of the tumor microenvironment, providing insights into mechanisms driving neoplastic cell migration and disease progression (3). Among chemokine receptors, CCR4 is considered the most pathogenetically and therapeutically relevant in MF/SS (26). In early-stage MF (patch/plaque lesions), CCR4 is expressed by both neoplastic T cells—located within the follicular epithelium and Pautrier microabscesses—and reactive T lymphocytes, indicating a role in skin homing. In tumor-stage MF, CCR4 is often co-expressed with CD30 on large atypical tumor cells infiltrating the dermis (3). Flow cytometric analysis of peripheral blood in tumor-stage MF has shown that clonal MF cells may constitute up to 50% of CD3^+^ lymphocytes. In one such case (patient 8, Kallinich study), high expression levels of CCR4 and CCR7, along with partial CXCR4 expression, were observed (3). Shono et al. found that CCR4 expression significantly correlated with prognosis: patients with high CCR4 expression had a notably lower survival rate compared to those with low or absent expression. However, expression patterns varied with disease stage: strong CCR4 staining was more frequent in advanced MF/SS, though a substantial proportion of CCR4-negative cases was still observed—68% in early-stage MF and 62% in advanced-stage disease (20). To address the limited sensitivity of conventional immunohistochemistry, Sugaya et al. utilized a more sensitive CCR4 detection kit, revealing CCR4 expression across all stages of MF, with higher levels in tumor-stage lesions (7). Kallinich et al. confirmed consistent CCR4 expression in MF skin lesions—both early and advanced—as well as in peripheral blood. However, one case of tumor-stage MF entirely lacked CCR4 expression (3). Campbell et al. demonstrated that most circulating CD4^+^ T cells expressing cutaneous lymphocyte antigen (CLA) also co-express CCR4, marking them as skin-homing T cells (17). These findings support the clinical utility of monoclonal antibodies targeting CCR4. Mogamulizumab, a humanized anti-CCR4 monoclonal antibody, received FDA and EMA approval in 2018 for the treatment of adults with relapsed or refractory MF/SS following at least one systemic therapy. In clinical practice, it is primarily used as a second-line agent in advanced-stage MF (IIB–IVB) and SS, with selective use in early-stage disease (IB–IIA) (27). The MAVORIC retrospective study assessed the real-world effectiveness of mogamulizumab across Europe. A *post hoc* analysis revealed overall response rates (ORRs) of 26% in stage III, 36% in stage IV, 16% in stage IIB, and 19% in stages IA/IIA. Compartment-specific ORRs were 42% in skin, 68% in blood, and 17% in lymph nodes (28, 29). Patients with erythroderma—defined as patch/plaque lesions covering more than 80% of body surface area—and those with blood involvement (B1 or B2) responded better to mogamulizumab compared to patients with tumor-stage MF (30–32). Reduced efficacy in tumor-stage MF may partly be attributed to absent CCR4 expression in certain patients, as illustrated by patient 12 in the Kallinich study (3).

### 2.2 C-C chemokine receptor type 7

#### 2.2.1 Physiological role of CCR7

C-C chemokine receptor type 7 (CCR7) is a G protein-coupled receptor predominantly expressed on naïve T cells, central memory T cells, mature dendritic cells, and subsets of B lymphocytes. It plays a crucial role in immune cell homing by directing the trafficking of these cells to secondary lymphoid organs—such as lymph nodes and the spleen—through interaction with its ligands CCL19 (also known as ELC—Epstein-Barr virus-induced molecule 1 ligand chemokine) and CCL21 (also known as SLC—Secondary Lymphoid Tissue Chemokine). CCL19 and CCL21 are produced by high endothelial venules and lymphatic endothelial cells (13, 33). This mechanism allows for efficient antigen scanning and the initiation of adaptive immune responses. Moreover, CCL19 and CCL21 are the two principal ligands of CCR7 and are members of the CC chemokine family. They are critical in guiding the migration of CCR7^+^ immune cells toward secondary lymphoid organs. CCL19 is primarily produced by fibroblastic reticular stromal cells in lymph nodes, while CCL21 is predominantly expressed by lymphatic endothelial cells (13, 33, 34). CCL21 possesses a highly charged C-terminal domain that binds glycosaminoglycans in the extracellular matrix, allowing it to form immobilized chemokine gradients. These gradients provide directional cues that guide the migration of CCR7^+^ cells, especially in the context of lymphatic entry and tissue compartmentalization. The complementary expression patterns and functional distinctions between CCL19 and CCL21 ensure coordinated lymphocyte homing, dendritic cell localization, and the spatial organization of immune responses within lymphoid tissues.

Beyond its migratory function, CCR7 is actively involved in immune synapse formation and intracellular signaling. Upon ligand engagement, CCR7 promotes T cell polarization and activation of key molecules such as ZAP70. It also modulates integrin affinity—particularly LFA-1—to stabilize interactions between T cells and antigen-presenting cells, facilitating T cell priming and the development of long-lasting immune memory. In the central nervous system (CNS), CCR7 regulates immune surveillance under both homeostatic and inflammatory conditions. It facilitates the migration of antigen-presenting cells and T lymphocytes between peripheral lymphoid tissues and the CNS. This function is essential for basal immunological monitoring and becomes critical during neuroinflammation, helping to coordinate targeted immune responses while minimizing collateral tissue damage (35, 36).

#### 2.2.2 CCR7 in mycosis fungoides and Sézary syndrome

In MF and SS, CCR7 expression is associated with disease dissemination and lymph node involvement. Normally expressed by central memory T cells, CCR7 marks a central memory-like phenotype in Sézary cells, enabling their migration through lymphatic vessels to secondary lymphoid organs (2, 37). This supports the widely accepted notion that SS arises from central memory T cells, whereas MF likely originates from skin-resident effector memory T cells, which typically lack CCR7 expression. However, emerging data suggest that CCR7 may be re-expressed in advanced MF, particularly in cases with dermal or subcutaneous tumor infiltration. In these scenarios, tumor cells often exhibit CCR7 positivity alongside increased extracellular matrix levels of CCL21, supporting enhanced migratory capacity mediated via the PI3K/Akt and mTOR signaling pathways (33, 38, 39). Functional assays using the CCR7^+^ MyLa MF cell line have demonstrated directional migration toward CCL21 and concurrent upregulation of the metastasis-associated lncRNA MALAT1, reinforcing the functional significance of CCR7 in disease progression (38). The variable expression of CCR7 among MF/SS subtypes reflects their biological heterogeneity. High CCR7 expression has been linked to nodal tropism, increased dissemination potential, and apoptosis resistance, underscoring its pathophysiological importance and highlighting its promise as a potential therapeutic target (37). Despite consistent findings regarding CCR7 expression in CTCL—particularly in SS and late-stage MF—its utility as a prognostic marker remains uncertain. Biologically, CCR7 contributes to lymph node homing, immune evasion, and cell survival, aligning with the clinical hallmarks of nodal dissemination and systemic disease progression. However, robust clinical evidence linking CCR7 expression to outcomes such as progression-free or overall survival is lacking. Most available data are derived from *in vitro* models, transcriptomic studies, or small patient cohorts, often without longitudinal clinical correlation. In early-stage MF, CCR7 is typically absent, whereas in advanced disease with deeper tissue infiltration, it may be expressed. Whether this reflects a shift toward a more aggressive malignant phenotype or an adaptive response to microenvironmental cues is unclear. Moreover, intralesional heterogeneity and dynamic regulation of CCR7 expression further complicate interpretation. While CCR7 holds considerable promise as a therapeutic target, current data do not support its routine use as a prognostic biomarker in clinical practice. Future prospective studies that combine molecular profiling with clinical follow-up will be critical to clarify its prognostic and predictive relevance.

### 2.3 C-C chemokine receptor type 8

#### 2.3.1 Physiological role of CCR8

CCR8 is a G protein–coupled chemokine receptor whose primary known ligand is CCL1 (4, 40). Physiologically, CCR8 is expressed on a range of immune cells, including Th2 lymphocytes, Tregs, skin-resident memory T cells, and subsets of monocytes and dendritic cells (41) and its expression is associated with immune regulation and tissue-specific immune cell recruitment, particularly in barrier tissues such as the skin and lungs.

CCR8–CCL1 signaling plays a role in maintaining immune tolerance, especially in tissues where Treg activity is essential to prevent autoimmune inflammation (42). In the skin, CCR8 is highly expressed by CD4^+^CD103^+^CD69^+^ skin-resident memory T cells, suggesting a role in long-term cutaneous immune surveillance and local memory responses (43, 44). CCL1 is produced by activated endothelial cells, keratinocytes, and myeloid cells during inflammation and facilitates the recruitment of CCR8-expressing T cells to sites of tissue injury or infection. Beyond its role in inflammation, this axis also contributes to tumor immunology, where it is involved in the accumulation of immunosuppressive Tregs within the tumor microenvironment (45).

#### 2.3.2 CCR8 in mycosis fungoides and Sézary syndrome

CCR8 has emerged as a key skin-homing receptor expressed on neoplastic T cells in MF and SS, and its involvement appears especially relevant in advanced stages of disease: it has been demonstrated that CCR8 is upregulated on malignant CD4^+^ T cells in SS and tumor-stage MF, contributing to their epidermotropism and persistence in skin (43, 44). Immunophenotyping analyses have confirmed CCR8^+^ neoplastic cells in lesional skin and in the circulation of SS patients, often co-expressing other skin-homing molecules such as CLA and CCR4, indicating a synergistic mechanism of cutaneous tropism (46).

The expression of CCR8 in MF/SS is associated with a Th2-prevalent cytokine environment, which favors tumor progression and immune evasion. IL-4 and IL-13, cytokines abundantly produced in later stages of MF and in SS, can enhance CCR8 expression on malignant T cells and may stimulate keratinocytes and myeloid cells to secrete CCL1, thereby creating a positive feedback loop for tumor cell recruitment and survival in the skin (39, 42, 43).

Importantly, CCR8 is not typically expressed in benign conditions and disorders, supporting its potential diagnostic utility in distinguishing MF/SS from mimickers (47, 48). Furthermore, due to its restricted expression pattern and its role in maintaining the immunosuppressive skin microenvironment, CCR8 has been proposed as a therapeutic target, particularly in strategies aiming to deplete Tregs or interfere with neoplastic T cell homing (45, 49–51).

Several monoclonal antibodies targeting CCR8 are currently under preclinical or early clinical investigation, aiming to selectively deplete CCR8^+^ tumor-infiltrating T cells or enhance anti-tumor immune responses; antibodies with Fc-effector functions have shown the capacity to eliminate intratumoral Tregs while sparing peripheral immune tolerance electively (50, 50, 52).

### 2.4 C-C chemokine receptor type 10

#### 2.4.1 Physiological role of CCR10

CCR10 is a chemokine receptor whose known ligands are CCL27 and CCL28. It is physiologically expressed by melanocytes, plasma cells, and skin-homing T cells, and plays a critical role in lymphocyte trafficking to cutaneous and mucosal tissues. CCR10 is expressed on subsets of αβ and γδ T cells, Tregs, innate lymphoid cells (ILCs), and IgA^+^ plasmablasts. Its function is tightly regulated by tissue-specific ligand expression, allowing selective immune cell migration. CCL27, also known as cutaneous T-cell–attracting chemokine (CTACK), is a skin-associated chemokine predominantly expressed by keratinocytes. It plays a pivotal role in the recruitment of CCR10-expressing T cells to the skin, thus maintaining cutaneous immune surveillance and homeostasis. By facilitating T cell localization to the epidermis and dermis under both homeostatic and inflammatory conditions, the CCL27–CCR10 axis is essential for skin-specific immune responses (53, 54).

This chemokine–receptor interaction is particularly relevant during inflammatory skin diseases, including psoriasis and atopic dermatitis, where aberrant T cell trafficking contributes to pathogenesis. Moreover, the skin-restricted expression of CCL27 has made it an attractive therapeutic target in immune-mediated cutaneous disorders, as interventions modulating this pathway may allow for site-specific immunomodulation with reduced systemic effects (55). CCL28, by contrast, is secreted by epithelial cells in mucosal tissues such as the colon and nasal mucosa, and guides the migration of IgA^+^ plasmablasts and memory T cells (56).

#### 2.4.2 CCR10 in mycosis fungoides and Sézary syndrome

CCR10 is expressed on malignant T cells in MF and SS (57), where it interacts with CTACK/CCL27—produced by basal and suprabasal keratinocytes—playing a key role in skin tropism. This skin-homing mechanism is active even in early stages of MF (14). Interestingly, the number of circulating CCR10^+^ T cells is significantly elevated in MF patients—including those at early stages—compared to healthy controls. However, CD4^+^CCR10^+^ T cell counts do not significantly vary between different MF stages. Serum levels of CTACK/CCL27 were found to be higher in patients with plaque or tumor-stage lesions than in those with patch-stage MF (14). This variation in CCL27 expression by keratinocytes appears to be influenced by the effects of Th1 and Th2 cytokines. Specifically, IFN-γ, the key cytokine of the Th1 response and predominantly expressed in early-stage MF, suppresses CCL27 expression in keratinocytes. Conversely, Th2 cytokines, particularly IL-4, enhance CCL27 expression and promote the recruitment of CCR10- and CCR4-positive T lymphocytes, which are characteristic of the advanced stages of MF (58, 59).

Hiraku et al. reported increased CCR10 expression in patients with erythrodermic CTCL, as compared to those with inflammatory dermatoses or non-erythrodermic MF. In contrast, primary CD30^+^ cutaneous lymphomas generally do not express CCR10 (20, 60). These observations highlight the role of the CCR10–CCL27/CCL28 axis in mediating cutaneous homing of neoplastic T cells in MF and SS, suggesting potential diagnostic and therapeutic implications (20, 23).

### 2.5 C-X-C chemokine receptor 3

#### 2.5.1 Physiological role of CXCR3

CXC chemokine receptor 3 (CXCR3) is a G-protein-coupled receptor (GPCR) classified as a CXC-type receptor based on the structural features of its ligands (61, 62). Unlike most chemokine receptor genes, CXCR3 is uniquely located on the Xq13 region of the human X chromosome (63). It is preferentially expressed in Th1-type activated/memory CD4^+^ T cells and binds three high-affinity ligands: CXCL9 (MIG), CXCL10 (IP-10), and CXCL11 (I-TAC), all of which are induced by IFN-γ (13, 61). Although originally thought to be restricted to activated T cells and NK cells (63, 64), CXCR3 is now known to be expressed on regulatory T cells (65), γδ T cells (66), B cells (67), and dendritic cells (68). Expression has also been reported in eosinophils (69), neutrophils (70), and several non-immune cells such as fibroblasts, epithelial and endothelial cells, astrocytes, and smooth muscle cells (61, 62, 68, 71, 72). The CXCR3–ligand system plays two main roles: recruitment of activated immune cells to sites of inflammation, and inhibition of angiogenesis. In inflamed tissues, an IFN-γ-driven positive feedback loop between Th1 cells and resident immune or epithelial/endothelial cells enhances CXCR3 ligand expression, promoting further recruitment of CXCR3^+^ effector cells (64, 71). CXCR3 also mediates recruitment of cytotoxic CD8^+^ T cells to infected or inflamed tissues (73). The anti-angiogenic function is attributed to the alternatively spliced CXCR3-B isoform expressed on endothelial cells, suggesting a potential antitumor role (71, 72). Moreover, CXCR3 expression on dendritic cells enables their migration toward secondary lymphoid organs in response to CXCL9 (68).

#### 2.5.2 CXCR3 in mycosis fungoides and Sézary syndrome

Jones et al. (74) demonstrated that CXCR3 is expressed in specific subtypes of peripheral T-cell lymphomas and contributes to chemotaxis in these malignancies. It is characteristically expressed in smaller neoplastic T cells of angioimmunoblastic T-cell lymphoma and angiocentric or histiocyte-rich T-cell lymphomas, while typically absent in ALK-positive anaplastic large-cell lymphoma (20, 60, 74). In MF, the expression of CXCR3 varies during disease progression. Early-stage MF (patch and plaque lesions) features small to intermediate-sized tumor cells that exhibit epidermotropism, and these tumor cells frequently express CXCR3 (75). Kakinuma et al. (15) reported CXCR3 positivity in both epidermotropic and dermal lymphoid infiltrates in early-stage lesions, but its expression declines or becomes absent in the tumor stage, particularly in large transformed neoplastic cells. Despite this, reactive T cells continue to express CXCR3 even in tumor-stage MF (3). CXCR3 is notably downregulated in erythrodermic cutaneous T-cell lymphomas, including Sézary syndrome. Since CXCR3 is typically upregulated in inflammatory dermatoses (e.g., atopic dermatitis), its downregulation in erythrodermic CTCL can aid differential diagnosis (20, 60). CXCR3 ligands are also implicated in MF pathogenesis. Sarris et al. (76) found that CXCL10 (IP-10) is overexpressed in MF lesional keratinocytes, extending beyond the basal layer into suprabasal keratinocytes. Tensen et al. (77) also detected CXCL9 (MIG) mRNA in epidermotropic cutaneous T-cell lymphomas. These findings support a model in which CXCR3 mediates lymphocyte epidermotropism via keratinocyte-derived CXCL9/10/11. As transformed tumor cells progressively lose CXCR3 expression, they become less responsive to epidermal chemotactic signals, contributing to loss of epidermotropism and dermal accumulation. Furthermore, reduced IFN-γ production by infiltrating lymphocytes likely leads to diminished CXCL9/10 expression in the epidermis (20, 76, 77). Importantly, Winter et al. (78) demonstrated that in advanced CTCL, CXCR3 surface expression and functionality are impaired on CD8^+^ cytotoxic T cells. This defect may prevent their infiltration into tumor sites, contributing to immune evasion and disease progression.

### 2.6 C-X-C chemokine receptor 4

#### 2.6.1 Physiological role of CXCR4

CXCR4 is a chemokine receptor that specifically binds to its unique ligand, CXCL12. This interaction plays a central role in lymphocyte chemotaxis toward sites of inflammation and also contributes to the migration of malignant cells (79). Under physiological conditions and in the context of inflammation, CXCR4 is widely expressed by various hematopoietic cells circulating in peripheral blood, including CD34^+^ progenitor cells, neutrophils, dendritic cells, B and T lymphocytes, and approximately 50% of peripheral T cells (3, 80).

#### 2.6.2 CXCR4 in mycosis fungoides and Sézary syndrome

Several studies have investigated CXCR4 expression in MF and SS, often with conflicting results. In early-stage MF with patch and plaque lesions, most malignant T cells express CXCR4, while only a minority of reactive T cells are positive. Similarly, in tumor-stage MF, high CXCR4 expression is seen in malignant CD3-deficient T cells, whereas only a small fraction of CD3^+^ inflammatory T cells express the receptor. CXCR4 was also found to be strongly expressed in epidermotropic infiltrates associated with epidermal erosion (3). In the study by Kallinich et al., partial CXCR4 expression was detected on circulating cells in one patient (Patient 8) (3). These findings suggest a possible synergistic role of CCR4 and CXCR4 in directing the skin homing of malignant T lymphocytes. An immunohistochemical analysis by Dagget et al. demonstrated increased CXCR4 expression in MF lesions compared to healthy skin, while also observing lower CXCR4 expression on neoplastic T cells than on reactive lymphocytes (81, 82). Furthermore, CXCR4 expression appeared stage-dependent: it was more pronounced in plaque-stage MF than in patch- or tumor-stage lesions, where expression was weak or absent. Similar trends were observed for the CXCL12 ligand. In contrast, Maj et al. reported no statistically significant differences in CXCR4 or CXCL12 expression between early and advanced MF, although both were upregulated relative to healthy skin (82).The CXCL12–CXCR4 axis is a well-established driver of tumorigenesis, supporting angiogenesis, invasion, and metastasis in various carcinomas (83). In CTCL, it may contribute to the skin-homing of Sézary cells via downregulation of CD26, a surface enzyme that physiologically cleaves and inactivates CXCL12.

Reduced CD26 expression enhances CXCL12-mediated chemotaxis of Sézary cells (84). Aronovich et al. found that fibroblasts within MF lesions exhibited greater proliferative capacity than those from normal skin. These cancer-associated fibroblasts (CAFs) protected MF cells from doxorubicin-induced apoptosis and enhanced their migratory ability by secreting CXCL12. This highlights the potential of targeting the CAF-driven microenvironment to improve therapeutic outcomes in MF (85). This mechanism has both pathogenetic and diagnostic significance, as reduced or absent CD26 expression is a criterion in the EORTC blood classification system for MF/SS (86).

Notably, Moyal et al. (2021) reported that CXCR4-expressing CAFs promoted migration and drug resistance in Mycosis Fungoides models, and that the CXCR4 antagonist plerixafor (AMD3100) enhanced apoptosis and reduced motility of neoplastic T cells *in vitro (* 87, 88); these findings support the concept that impairing the CXCL12/CXCR4 axis may have direct anti-tumor effects beyond its established role in stem cell mobilization (89).

Although plerixafor is approved for hematopoietic stem cell mobilization in lymphoma, multiple preclinical studies in other hematologic and solid tumors, including leukemias and diffuse large B-cell lymphoma, have shown that CXCR4 blockade can synergize with chemotherapy, sensitize tumor cells, and modulate the altered tumor microenvironment (82). Taken together, these data suggest a possible novel therapeutic rationale for exploring drugs altering this axis as an adjunctive treatment in CTCL, justifying further mechanistic studies and clinical evaluation.

Importantly, the histone deacetylase inhibitor vorinostat has been shown to downregulate CXCR4 expression in MF cell lines, suggesting a potential therapeutic approach to modulate chemokine receptor-mediated tumor progression (90).

### 2.7 Physiological Role of CCR1, CCR2, CCR3, CCR5, CCR6, CXCR1, CXCR2, CXCR5, and CX3CR1

Due to the limited evidence supporting a role for CCR1, CCR2, CCR3, CCR5, CCR6, CXCR1, CXCR2, CXCR5, and CX3CR1 in the pathogenesis or progression of MF and SS, their physiological functions will be briefly summarized. CCR1 and CCR5 are involved in mediating the recruitment of monocytes and T cells to sites of acute and chronic inflammation (91). CCR2 plays a pivotal role in the mobilization of monocytes from the bone marrow and is central to the perpetuation of chronic inflammatory processes (92). CCR3 primarily regulates eosinophil chemotaxis and activation and is associated with allergic inflammation (93), while CCR6 directs immature dendritic cells and memory T cells to epithelial surfaces, contributing to mucosal immunity (94). CXCR1 and CXCR2 are both receptors for interleukin-8 (IL-8) and are crucial for neutrophil chemotaxis and activation during inflammatory responses (95). CXCR5, while not implicated in skin homing, is critical for B cell migration into lymphoid follicles and germinal center organization. CX3CR1 functions primarily in monocyte and microglia trafficking and has been implicated in neuroinflammation and maintaining vascular integrity, particularly in non-cutaneous tissues (96).

#### 2.7.1 CCR1, CCR2, CCR3, CCR5, CCR6, CXCR1, CXCR2, CXCR5, and CX3CR1 in mycosis fungoides and Sézary syndrome

The roles of CCR1, CCR2, CCR3, CCR6, CXCR1, CXCR2, CXCR5, and CX3CR1 in CTCLs have been investigated, but findings indicate that these chemokine receptors play a minimal or no role in the pathogenesis of MF/SS. In a study by Kallinich et al. (3), immunohistochemical analyses revealed an absence of expression of these chemokine receptors in MF lesions. Expression in reactive non-neoplastic T cells was marginal at best, further suggesting a lack of functional relevance of these receptors in MF/SS pathogenesis.

As such, current evidence does not support a pathogenic role for these receptors in MF/SS, and no subsequent studies have challenged or expanded upon these findings.

An exception may be CCR5, for which limited reports suggest a potential contribution to disease progression. Berahovich et al. (97) reported that CCR5 may be upregulated in malignant T cells in MF, potentially enhancing their ability to migrate into skin regions enriched in CCR5 ligands—namely, CCL3, CCL4, and CCL5. This interaction may promote the accumulation of neoplastic T cells in lesional skin and foster a pro-inflammatory tumor microenvironment. Such a scenario could facilitate immune evasion and chronic inflammation, potentially contributing to disease progression and immunosuppression.

However, due to the paucity of corroborating studies, CCR5’s precise role in MF/SS remains uncertain and warrants further investigation.

## 3 Discussion

Chemokines constitute a large family of polypeptide signaling molecules that play a central role in directing the chemotaxis of various cell types. They are responsible for guiding directional migration, mediating leukocyte homing, and exert their effects primarily through G protein-coupled receptors (GPCRs). The involvement of chemokines is well documented across a broad spectrum of pathological conditions, including benign, inflammatory, and neoplastic diseases (7, 10, 98). In oncology, chemokines contribute significantly to tumor progression and metastasis (5, 74, 83). Their role is particularly relevant CTCL, especially in MF and SS, where they are critically involved in disease progression from early to advanced stages, and in directing neoplastic cell homing to the skin, bloodstream, and metastatic sites (23, 60). Some chemokines are more highly expressed as the disease progresses to advanced stages. In particular, the receptors CCR4 and CCR7 show increased expression in advanced stages of the disease. Activation of the CCL17–CCR4 axis is associated with disease progression and the development of a more immunosuppressive microenvironment.

Specifically, serum levels of CCL17 are elevated in advanced-stage MF and SS. CCL17, produced by cells in the tumor microenvironment, contributes to the recruitment of Th2-polarized malignant T lymphocytes Tregs. CCR7 is typically expressed in SS, and its levels are also elevated in advanced-stage MF, particularly in cases with tumor lesions and lymph node involvement. CCR7 interacts with its ligand CCL21, which is found at higher levels in the extracellular matrix of tumor lesions. By contrast, CCR7 expression is low in early-stage MF. Conversely, expression of CXCR3 and its ligands is higher in early-stage MF, where lesions are characterized by epidermotropic neoplastic infiltrates. In advanced stages, large transformed neoplastic cells in the dermis are typically negative for CXCR3. For other receptors, findings vary considerably between studies. For example, CCR10 expression does not appear to change significantly with disease stage (14), although CCL27 levels are lower in patch lesions compared to plaque and tumor-stage lesions. Other studies, however, suggest that CCR10 is more highly expressed in erythrodermic CTCLs (20, 60). Similarly, the relationship between CXCR4 expression and disease stage remains controversial. Daggett et al. observed higher expression of CXCR4 and its ligand CXCL12 in plaque lesions than in patch or tumor lesions, whereas Maj et al. found no significant differences between early and advanced-stage MF (82).

The study of chemokines has also helped clarify the origin of neoplastic clones in MF and SS. SS cells are characterized by the expression of CCR7. This receptor is typically found on central memory T cells and directs their chemotaxis toward lymph nodes via the lymphatic vessels. In contrast, MF cells do not express CCR7 and instead display a phenotype consistent with skin-resident effector memory T cells.

The expression profiles of various chemokine receptors and their corresponding ligands help delineate the progression from early to advanced stages of CTCL. Importantly, assessing these expression levels—when appropriately integrated with clinical and histopathological data—may aid in the differential diagnosis between inflammatory skin diseases and early or erythrodermic MF. Indeed, distinguishing CTCL from benign inflammatory dermatoses remains a persistent challenge in clinical practice and is often associated with the risk of misdiagnosis and inappropriate treatment (3, 11, 15, 81, 99). For further details, see **Tables 1**, **2**.

**Table 1:** Expression of chemokine receptors and their ligands in early and advanced CTCL.

Receptor	Ligands	Expression in early MF	Expression in advanced MF and SS
CCR4	CCL22 and CCL17	Expressed by both neoplastic T cells and reactive T lymphocytes (Kallinich et al.).	High CCR4 expression is more frequent in advanced MF and SS (Shono et al., Sugaya et al.).
CCR7	CCL19 and CCL21	Typically absent (Campbell et al., Kallinich et al.).	Expressed (Campbell et al., Kallinich et al.).
CCR8	CCL1	limited expression in reactive T cells (Guistiniani et al., de Masson et al., Geller et al.)	Higher prevalence in advanced MF and SS; proposed as diagnostic and therapeutic biomarker (Geller et al., Guistiniani et al.).
CCR10	CCL27 and CCL28	Elevated (Fujita et al.).	Not vary between different early and advanced-MF (Fujita et al.)
CXCR3	CXCL9, CXCL10 and CXCL11	Highly expressed on neoplastic and reactive cells (Kakinuma et al., Lu et al., Nikoo, Kallinich et al.).	Not expressed in neoplastic T cells, but expressed on reactive T cells (Kallinich et al., Lu et al.). Downregulated in erythrodermic-stage MF and in SS (Shono et al., Suga et al.).
CXCR4	CXCL12	Expressed on most neoplastic T cells express CXCR4, but not on reactive T cells (Kallinich et al.).	Higher in malignant T-cells than in inflammatory T cells (Kallinich et al.).

**Table 2:** Functional role and prognostic value of chemokine receptors and their ligands in cutaneous T-Cell lymphoma.

Receptor	Ligands	Functional Role and prognostic value
CCR4	CCL22 and CCL17	• Skin-homing of neoplastic cells (Pileri et al., Shono et al.). • Decreased survival rate (Shono et al.). • Correlation with elevated LDH serum levels and increased disease activity in MF/SS (Kakimura et al.). • Therapeutic use of Mogamulizumab in advanced-MF/SS (Ollila et al.).
CCR7	CCL19 and CCL21	• Association with systemic disease progression • Migration through lymphatic vessels to secondary lymphoid organs (Wu et al., Campbell et al.).
CCR8	CCL1	• Skin-homing of neoplastic T cells, especially in advanced-stage MF and SS (Guistiniani et al., de Masson et al., Geller et al.). • Proposed as a diagnostic and therapeutic biomarker (Geller et al.). • Targetable by monoclonal antibodies; potential for reduced immune-related adverse events compared to anti-CCR4 therapy (Silva et al., de Masson et al.).
CCR10	CCL27 and CCL28	• Skin-homing of neoplastic cells (Fujita et al., Shono et al., Sokolowska-Wojdylo et al.).
CXCR3	CXCL9, CXCL10 and CXCL11	• Potential antitumor role given its anti-angiogenetic function (Lacotte et al., Billottet et al.). • Associated with epidermotropism (Sarris et al., Tensen et al., Lu et al., Shono et al.).
CXCR4	CXCL12	• Involved in tumorigenesis by angiogenesis, invasion, and metastasis (Balkwill et al.). • Skin-homing of neoplastic cells, in particular Sézary cells (Narducci et al.)

**Figure 1:**
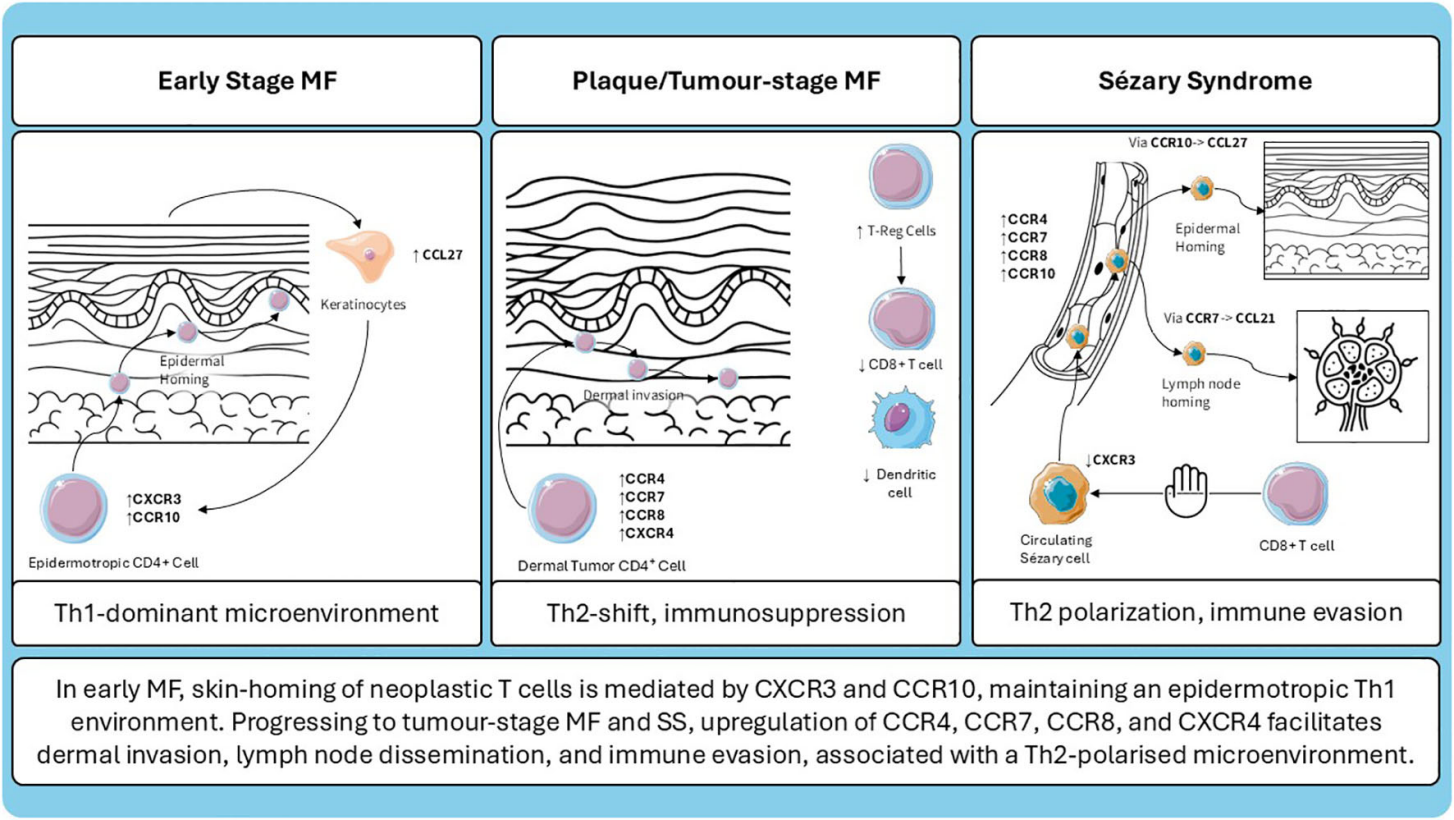
Schematic representation of chemokine receptor expression and microenvironmental changes across different stages of cutaneous T-cell lymphoma.

The mechanisms involved in the progression form early lesions to advanced disease in MF and SS are complex and not fully understood. Many players are involved and one of them is chemokines and among them CCR4 can be defined as the “lion queen”; schematic summary of the evolving chemokine receptor landscape and microenvironmental shifts in MF and SS is presented in (**Figure 1**). To date is the one that have most therapeutic implications because of the availability of the anti-CCR4 mogamulizumab. For sure, as observed by Ohuchi et al. (100) in Mogamulizumab-responder patients, decreased serum levels of CCL22 are related to a better response. CCL22 is overexpressed in M2 macrophages surrounding neoplastic cells in MF infiltrates (100). One possible explanation of Mogamulizumab action may be the reduction of immunosuppressive M2 macrophages, inducing the awakening of host immune system against the disease, stressing the concept that Mogamulizumab action is not merely focused on neoplastic T-cells but also to a switch from a Th2 immune suppressive to a Th1 anti-tumor microenvironment. The ability of refractory/relapsed disease in Moga-treated patients is related to the loss of CCR4 expression a finding that again reinforce the pivotal role of CCR4 in the treatment of the patients (101) more than its receptors CCL17 and CCL22. Another intriguing cytokine receptor is CCR5 that by recruiting neoplastic cells within lesional skin may prompt disease progression increasing the levels of immune-suppressive cytokines secreted by tumor cells, that a downstream may lead to the recruitment of immune suppressive cells (as immature CD209+ dermal dendritic cell, myeloid derived suppressor cells) (21). All the above mentioned concepts underline how many players (tumor cells, microenvironment, cytokines, chemokines) are involved and are connected among them. The knowledge of all the interaction between players involved in MF pathogenesis and progression is ultimately important to develop new therapies in MF and SS.

## 4 Conclusion

Investigating the differential expression of chemokine receptors and their corresponding ligands in CTCL has provided valuable insights into the interactions between neoplastic cells and the various components of the tumor microenvironment. Moreover, analyzing chemokine receptor expression has made it possible to clearly distinguish the cellular origin of neoplastic clones in CTCL, effectively differentiating MF from SS. In addition, chemokine expression profiles differ between CTCL and benign inflammatory dermatoses.

Further studies on chemokines and their receptors in CTCLs will enhance our understanding of the pathogenic mechanisms underlying disease progression. Most importantly, they may offer a biological rationale for the development of novel targeted therapies.

This knowledge enhances our understanding of the biological behavior of the disease and may help identify potential diagnostic markers and therapeutic targets.
